# Unpacking the Potential Influence of Life Satisfaction on Network Heterogeneity, Emotional Exhaustion and Mobile App Fatigue: A Stressor–Strain–Outcome Approach

**DOI:** 10.3390/ijerph20043500

**Published:** 2023-02-16

**Authors:** Hua Pang, Qinglong Shao

**Affiliations:** 1 School of New Media and Communication, Tianjin University, Tianjin 300072, China; 2 Institute of Chinese Studies, Freie Universität Berlin, 14195 Berlin, Germany

**Keywords:** life satisfaction, upward comparison, self-presentation, emotional exhaustion, mobile app fatigue

## Abstract

The ramifications of mobile apps’ detrimental aspect on users’ life satisfaction have garnered increased attention from academics. To probe the underlying association between life satisfaction and mobile app fatigue, this article builds a research model based on a stressor–strain–outcome approach. In addition, the study investigates the relationships between different dimensions of network heterogeneity, emotional exhaustion, and mobile app fatigue among users. Furthermore, the study uncovers the moderating influence of upward comparison, self-presentation, and privacy invasion on the association between life satisfaction and emotional exhaustion in the mobile app context. The study collected data in mainland China using a cross-sectional approach and analyzed the data using structural equation modeling. The findings demonstrate that life satisfaction is positively associated with self-presentation and negatively associated with upward comparison. Moreover, privacy invasion and upward comparison are positively correlated with emotional exhaustion, whilst self-presentation exerts no correlation with emotional exhaustion. Furthermore, upward comparison could mediate the association between life satisfaction and emotional exhaustion. The results provide fresh light on the mechanisms through which the life satisfaction of mobile app users and network heterogeneity might lead to emotional exhaustion and mobile app fatigue, highlighting important theoretical and practical implications.

## 1. Introduction

Recently, mobile applications (apps) have become increasingly embedded in contemporary society, with billions of worldwide active users devoting considerable time and energy to these platforms for various objectives, such as interacting with others and glancing over shared content [1,2,3]. Scholars have determined that mobile apps have assisted users in expanding their social contacts, improving their self-esteem and well-being, and benefiting from an abundance of options for self-presentation [4,5,6]. Numerous studies have explored the negative influences of mobile device usage on people’s well-being, including the potential for cyber stalking [7], fear of missing out [8,9], and mobile-app-use-induced adverse reactions, such as guilt [10], anxiety, depressive moods [11,12,13], and even exhaustion [3,14]. Users’ life satisfaction is one of the concerns that academics have been paying close attention to [15,16,17]. Scholars have conducted substantial research on life satisfaction in real-world situations using individuals’ overall appraisal of current life, depending on criteria selected by themselves.

Researchers have claimed that life satisfaction is a major factor in the popularity of any website or mobile app site [18,19]. This could be due to the truth that mobile apps promote user participation with a variety of interactions and communications, which results in advantages such as an increased gratification with web-based communication and creates bonding and bridging capital. Some research has uncovered the positive impacts of life satisfaction on various variables, including social capital, self-disclosure, and mental health [20,21,22]. Comparatively, researchers have seldom investigated how life satisfaction influences self-presentation, upward comparison, privacy invasion, and the drawbacks of mobile app usage. Over the last decade, there has been an increase in empirical studies aimed at understanding the detrimental aspect of mobile devices, which refers to the phenomena related to the mobile app use that negatively impacts individuals’ well-being, such as emotional exhaustion and mobile app fatigue [12,14,19].

In addition, the possible association between life satisfaction and the negative aspects of mobile apps has not been thoroughly studied. Mobile apps offer members a variety of benefits, such as chances to exchange information and express themselves [9,23,24]. Subsequently, these benefits would be linked to life satisfaction, such as the gratification that people obtain from their lives on social media by establishing a favorable self-impression and social connectivity [19,23,25]. As a result, it is reasonable to suppose that life satisfaction is one of the primary factors driving people to utilize mobile apps. The vital topic of whether it causes a downward cycle that results in users experiencing the dark side of mobile apps is still important and understudied. Furthermore, research suggests that individuals’ experiences with the negative aspects of mobile apps are influenced by intervening factors such as self-disclosure and upward comparison, which may be related to the types of activities or interactions they participate in on these services [1,2]. To provide additional insights into mobile app fatigue, the paper introduces network heterogeneity from the psychosocial standpoint and investigates how these structural features directly influence emotional exhaustion, as well as indirectly through the mediation of several variables.

According to the latest report, the number of mobile app users in mainland China surpassed 100 million in June 2018, with active users spending an average of 20.17 min per day on their devices [26,27]. In addition, among the users of mobile social apps, approximately 83% of them are between the ages of 18 and 34 [28]. Considering the exponential growth of mobile apps, the study employs the stressor–strain–outcome classical paradigm to address the critical antecedents of emotional exhaustion and mobile app fatigue among the mobile app users in mainland China. To fill these gaps, the study poses and responds to the two research questions: (1) What correlations exist between life satisfaction, self-presentation, upward comparison, private invasion, emotional exhaustion, and mobile app fatigue? (2) Do privacy invasion, self-presentation, and upward comparison mediate the relationship between life satisfaction and emotional exhaustion? By adopting this stressor–strain–outcome theoretical framework, the article attempts to comprehensively and systematically comprehend how life satisfaction and individual variations in the network heterogeneity-related features of privacy invasion, upward comparison, and self-presentation may result in the unfavorable effect of fatigue. The study further investigates how these network heterogeneity elements play the meditating role in the association between satisfaction with life and emotional exhaustion in the mobile app context. Findings from this empirical research may help mobile app service providers to improve the life satisfaction of their current and future users; hence, this reduces the likelihood of emotional exhaustion and fatigue. As a result, insights on life satisfaction’s associations with the negative aspects of mobile apps are important to practitioners who engage with people who exhibit symptoms of worse mental health and fatigue because of mobile apps. Such an understanding might assist people to better manage their online social connections and guide appropriate use practices to avoid harmful mobile app outcomes.

## 2. Theoretical Framework and Hypotheses Development

### 2.1. The Stressor–Strain–Outcome Theoretical Paradigm

The stressor–strain–outcome (SSO) theory is a prominent paradigm for connecting stressors and outcomes, and it is a critical research foundation for comprehending the stresses imposed by technologies [5,29,30]. This model has been employed in certain research to explain the influence that inner stress has on the professional performance of employees working in companies [12,31]. Similarly, it has been used in studies of user behavior in internet and technology systems to provide insight into the effects of online-related stress [29,32]. The SSO framework comprises three primary components, which are listed below. Stressor refers to the element that generates stress, also known as the stimulus, that has an influence on an individual’s behaviors and emotions, such as the qualities of communication services [29]. Strain is a term that describes the psychological changes that may occur in people as a result of the perceived stress [5]. Outcome relates to the changes in behavior that emerge from people’s psychological reactions, such as avoiding messages and being exhausted by new media [12,31].

Prior research has suggested that psychological well-being could offer technical assistance for individuals’ online communications and relations management, but could create adverse issues [29,32]. Life satisfaction reflects a wide range of feelings and emotions, such as the negative and positive effects experienced by internet and mobile app users. Researchers have asserted that life satisfaction is critical to the success of digital platforms, including mobile apps [33,34]. However, researchers have rarely studied the role of life satisfaction as a vital predictor of the distinct dimensions of network heterogeneity and the negative aspects of mobile app use, i.e., the dark aspect of mobile apps. The last decade has witnessed an increase in the number of empirical studies designed to examine the dark aspect of media, which refers to the phenomena related to smartphone use that have a negative impact on users’ emotional health, such as social media exhaustion [34,35,36]. Thus, more research is urgently required to verify the sophisticated relationships between the individual, technological, and situational factors that produce negative outcomes. Consequently, we assert that it would be advantageous to comprehend how life satisfaction influences the distinct dimensions of the network heterogeneity of users and results in negative outcomes. According to the existing literature on strain in the computer-mediated context [3,37], the study concentrates on three specific aspects of the technological strains related to life satisfaction: self-presentation, upward comparison, and privacy invasion. In this research, negative concerns may cause users to have negative psychological responses and behavioral effects, such as emotional exhaustion or mobile app fatigue. As a result, emotional exhaustion and mobile app fatigue are included as the final outcome factors in this research. In addition, the research investigates whether self-presentation, upward comparison, and privacy invasion could attenuate the impact of life satisfaction on negative consequences.

### 2.2. Linking Life Satisfaction to Self-Presentation and Upward Comparison

As a key indicator of well-being, life satisfaction refers to how an individual evaluates his or her life during the majority of the time, or for a certain period of time [16,18,38]. Numerous personal, psychological, behavioral, interpersonal, and social consequences have been demonstrated to be favorably correlated with it. When individuals have a high-quality social network and experience additional social support from such networks, they typically have greater life satisfaction. According to some experts, self-presentation on mobile apps generates various benefits, including fostering friendships and intimate relationships, engaging in self-expression, and establishing one’s own identity [15,16]. Self-presentation, a widespread phenomenon within interpersonal interaction, is the conscious act of impression management through which individuals seek to construct, alter, or preserve their reputation in the view of other people. Fan et al. asserted that a person’s self-perception is dependent on the interaction goals and anticipations of interpersonal networks [16].

In terms of the linkage between life satisfaction and self-presentation, psychological state has been consistently discovered to be positively related to an individual’s self-presentation [17,18,30]. For instance, Grieve and Watkinson demonstrated that inauthentic self-presentation on mobile services can be related to numerous psychological problems of maladjustment [17]. Similarity, Fan et al. determined that well-being was negatively impacted because of how others presented themselves on social media platforms [16]. More recently, Tian et al. confirmed that online self-presentation was negatively connected with the life satisfaction among college students [18]. While there is no existing evidence for the influence of life satisfaction on self-presentation, the study asserts that mobile app users who have a high level of life satisfaction and experience positive emotions are more prone to be involved in self-presentation on mobile app services. The hypothesis is derived from previous research indicating that a person’s psychological state affects their self-presentation. Drawing conclusions from previous studies, the article proposes that mobile app users with a higher sense of life satisfaction may engage in more self-presentation practices to maintain their well-being and receive benefits such as pleasure and connection. In the mobile app context, the study thus anticipates that life satisfaction will positively correlate with self-presentation, since people use such newly burgeoning services to preserve their connectedness, relationships, and psychological well-being.

Upward comparison is defined as an individual’s comparison of oneself with others who are better off or superior than the comparer, which adversely affects the well-being of these individuals and lowers their self-regard [27,39]. Upward comparison is a typical psychological phenomenon that may have a significant influence on an individual’s sense of self-worth and their capacity for psychosocial adjustment [2,11]. A large number of prior studies have shown that upward social comparison may have an impact on people’s mental health and generate more unpleasant feelings, which is a risk factor for depressive moods [2,19,40]. Mobile apps are more than an acquaintance-based kind of network media. Individuals may display more favorable personal information on mobile apps to regulate their own impression and preserve positive self-images [32,41]. Existing evidence suggests that upward social comparisons might have a detrimental impact on people’s mental well-being and health [4,11]. According to the social rank theory of depression, sentiments of subordination or inferiority play a crucial role in the formation of negative tendencies in individuals [11]. Previous research relating social comparison to the many aspects of life satisfaction lends support to this supposition [4,15,16]. For example, Park and Baek suggested that the inclination of social media users to engage in upward comparison may have a detrimental impact on them by prompting them to experience harmful sentiments [4]. Recently, Yue et al. discovered that an individual’s upward social comparison orientation is related to their overall life happiness through the feelings elicited by such comparison [15]. Based on previous investigations, the study hypothesizes that negative emotions might well be associated with a greater propensity to engage in upward comparison. By comparing themselves to others on mobile app platforms, people would have greater resilience toward affirming their life satisfaction. Consequently, the study postulates the following assumptions:

**H1:**  *Life satisfaction is positively related to self-presentation.*

**H2:**  *Life satisfaction is negatively related to upward comparison.*

### 2.3. Linking Life Satisfaction to Privacy Invasion

According to previous research, a person’s most primary concern while utilizing mobile devices might be an invasion of their privacy [14,42]. The term privacy invasion refers to the perception that an individual’s privacy is being invaded as a consequence of this inappropriate usage of personal information by social media platforms [37]. Although a number of studies have found a correlation between privacy concerns and the amount of time spent on mobile app services, very few studies have been carried out to investigate the direct linkage between levels of life satisfaction and privacy invasion [3,21,43]. For instance, Dhir et al. discovered that privacy-related issues, such as self-portraits or selfies, are often taken and shared on computer-mediated systems, which is important for people’s psychological and emotional health in the modern world [43]. Durnell et al. revealed that when people’s privacy is breached, or their data is improperly managed, it may have far-reaching effects, both practically and emotionally [42]. Scholars have also claimed that particular affordances of mobile apps can also contribute to users’ feelings of insecurity regarding their personal information [3,14,44].

Additionally, the current study has uncovered the possible impact of users’ emotional states and sentiments about mobile devices and other new media applications on their privacy invasion and subsequent behavior [37,45,46]. For example, Kehr et al. suggested that online users tend to underestimate the dangers of information exposure when presented with a user interface that generates a positive effect, indicating that immediate emotional states impact privacy assessments [45]. Additional studies have established that people’s privacy-related judgments are founded on a complex web of interrelated logical and emotional perceptions, generated in the course of privacy-related circumstances [46]. A recent longitudinal investigation showed that users favored technologies including the Internet of things and smart-connected objects because they were pleased with their privacy features, which alerted them if their information was being duplicated, and their ability to save content for later viewing [21].

The degree of users’ observed privacy concerns may be linked to the satisfaction with life offered by their mobile apps, as well as the pleasant feelings connected to using these platforms [44,45]. However, until now, there has been no evidence supporting the claim that life satisfaction impacts users’ privacy invasion in the mobile app context. Indeed, incidents such as the trade of users’ information and the unauthorized extraction and utilization of private content by virus attacks or hackers, as well as other occurrences, have frequently infuriated people over privacy concerns [42,43]. Consequently, the more people’s understanding and evaluation of the severe risks connected with privacy breaches increases, the lower their degree of satisfaction with current life. Based on these observations, the article proposes the following hypothesis:

**H3:**  *Life satisfaction is negatively related to privacy invasion.*

### 2.4. The Mediating Role of Self-Presentation, Upward Comparison and Privacy Invasion

The three-element paradigm of burnout (psychological fatigue, depersonalization, and diminishing individual achievement) defines the experience of being emotionally exhausted and drained in performance [37,47]. It is distinguished by physical exhaustion, as well as a sense of being mentally and emotionally depleted. According to some scholars, the fundamental meaning of burnout is explained by the physical or psychological tiredness connected with emotional exhaustion [20]. Users’ mental, physical, and emotional reserves are exhausted as a result of the overwhelming amount of data generated by social media [37]. Although the majority of the research has explored the negative aspects of social media and regarded self-presentation as an antecedent of social media use-related outcomes, such as exhaustion, very few researchers have uncovered the indirect effects of self-presentation. For instance, self-presentation was shown to be a mediator between extroverted personality characteristics and social media usage [20,47]. Tian et al. further discovered that self-presentation could mediate between psychological state and positive aspects [18].

In addition, a handful of researchers have explored the mediating function of people’s tendencies to engage in self-comparative behavior when utilizing mobile devices [40,48,49]. Vogel et al. discovered that Facebook users’ attitudes toward social comparison moderated the relationship between neuroticism and the inactive usage of a certain social media site [40]. Likewise, Pedalino and Camerini determined that body appreciation decreases while using Instagram, and this effect is mediated only by upward comparison with social media influencers, rather than any other kind of peer [48]. Additionally, scholars have posited that the users of social media may engage in excessive passive usage of these platforms to gather knowledge, thus establishing a standard for upward comparison [49]. These studies suggest that those who have increased life satisfaction, especially people who are content and pleased with their lives on mobile apps, are more likely to feel the need to evaluate themselves in relation to those they encounter on these sites [45,49].

Furthermore, the potential influence of privacy invasion on mobile apps and related behavioral consequences has also been understudied from the perspective of exhaustion. Nonetheless, there is evidence in the literature that suggests privacy invasion could moderate the effects of self-presentation in the computer-mediated context. For example, based on a longitudinal analysis, Tsay-Vogel et al. found that the connections between Facebook usage and online disclosure could be mediated by privacy protections [50]. Later, Ioannou et al. determined that privacy concerns mediate the linkage between mindfulness and online UK individuals’ information disclosure [51]. More recently, Liu and Wei suggested that the influence of the open data policy on participants’ disclosure was mediated by their privacy-related invasion [52]. To the best of our knowledge, no available evidence supports the role of privacy invasion in moderating the association between life satisfaction and emotional exhaustion. This is a major gap in the research, since privacy issues play a large part in the negative aspects of mobile media-related concerns such as emotional exhaustion [40,42]. Therefore, the study hypothesizes the following:

**H4:**  *Self-presentation mediates the linkage between life satisfaction and emotional exhaustion.*

**H5:**  *Upward comparison mediates the linkage between life satisfaction and emotional exhaustion.*

**H6:**  *Privacy invasion mediates the linkage between life satisfaction and emotional exhaustion.*

### 2.5. Linking Emotional Exhaustion to Mobile App Fatigue

Generally, research considers fatigue to be a sort of psychological exhaustion and interprets it from the perspective of a stress-related phenomenon [8,53]. In the mobile app age, some scholars have characterized fatigue as a subjective, multi-faceted user experience comprising negative sentiments including exhaustion, frustration, poor interest, and diminished motivation, related to the different levels of mobile app usage [5,10]. Accordingly, the study describes mobile app fatigue as a continuum of the subjectively unpleasant experiences of weariness, depletion, strain, and lack of interest in response to operations. Previous research has focused primarily on various psychological frameworks to explain mobile app fatigue, consisting of the person–environment fit theory, the situation–organism–behavior–consequence paradigm, and the stimulus–organism–response model [1,53,54].

When the effects of user-related elements are considered, mobile apps exert a greater influence on users’ privacy and private information than their abilities to manage them [43,44]. Furthermore, users’ emotional management may become unsteady if their privacy is repeatedly violated [55]. The SSO paradigm posits that emotional fatigue is more likely to emerge in situations when one experiences a loss of possible resources, an inability to satisfy media demands, or an expectation of threats to the present resources [32]. When users do not believe they have the emotional resources to deal with the stress they encounter on mobile apps, they might experience prolonged strain or emotional exhaustion. In particular, the SSO hypothesis anticipates that people would feel uneasy and try to mitigate the loss. Because of this, scholars may explore the effects of mobile app fatigue on users’ actions and interactions. According to some investigations, individuals who are emotionally fatigued overemphasize avoidance or withdrawal as a coping mechanism [8,10]. According to the SSO theory, the article anticipated that emotional fatigue could positively predict mobile app fatigue.

**H7:**  *Emotional exhaustion is positively related to mobile app fatigue.*

## 3. Study Methodology

### 3.1. Study Model

**Figure 1:**
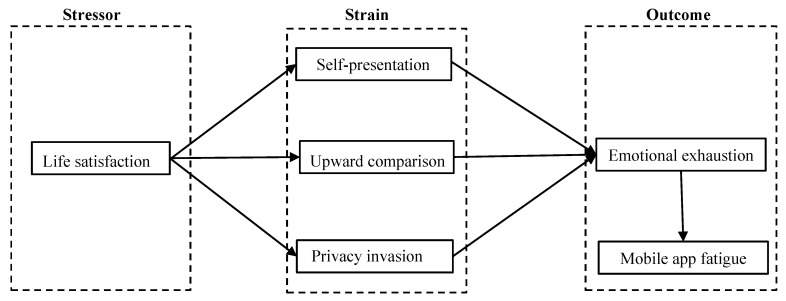
The proposed conceptual research model.

In this article, the stressor–strain–outcome (SSO) paradigm was employed to better understand the possible association between life satisfaction and fatigue in the context of mobile apps. Using the stressor–strain–outcome paradigm as a foundation, the article hypothesizes that individuals’ life satisfaction is associated with their perceived emotional exhaustion and fatigue. In addition, it is expected that self-presentation, upward comparison, and privacy invasion would mediate the relationship between life satisfaction and its associated emotional exhaustion. The proposed conceptual model, which depicts the hypothetical connection between the key variables, is illustrated in Figure 1.

### 3.2. Sample and Data Collection

During the months of October and November of 2022, the study gathered empirical data using a web-based survey hosted on wjx (http://www.wjx.cn accessed on 1 September 2022). The study distributed an invitation message on numerous mobile app groups in mainland China, followed by a link to the online questionnaire and an invitation to complete the survey. Eligible participants had interacted with others through mobile apps during the previous week. Participants were guaranteed that their responses would be kept confidential and utilized exclusively for study aims via a conspicuous statement on the questionnaire. The study received ethical approval from the Ethics Committee of Tianjin University. This research was confined to Chinese mobile app users, and data collection lasted around one month, with a total of 970 qualified users replying. After eliminating the invalid and incomplete responses, 866 valid questionnaires were utilized for further analysis.

### 3.3. Measurement

To assure the validity of the instrument, all the measurement items were acquired from prior research and assessed using multiple-item scales. This online survey was specifically prepared in two sections. The first section of the research consisted of variables: life satisfaction, self-presentation, upward comparison, privacy invasion, emotional exhaustion, and mobile app fatigue. The second section included demographic information from participants such as their gender, age, educational background, monthly income, and mobile app usage experience. To further validate the validity of this questionnaire, a pilot test was performed on 30 mobile app users at the start of the large-scale questionnaire survey. Based on the experimenters’ feedback, the study made several changes to enhance the clarity and understandability of the online survey.

#### 3.3.1. Life Satisfaction

This three-item scale was restructured from previously published research to better indicate participants’ satisfaction with life [18,56]. Sample items included: “In most aspects, my life is near to perfect”, and “My living circumstances are ideal”. All phrases were constructed utilizing a 5-point Likert-type scale (1 = strongly disagree, 5 = strongly agree). The Cronbach‘s alpha was 0.918, indicating a high reliability (M = 3.55, SD = 1.02).

#### 3.3.2. Self-Presentation

In total, three measures from a previous study on self-presentation were used in this investigation [1]. Certain items included the following statements: “I have a thorough mobile app profile,” and “I expose a great deal about myself on mobile app.” To evaluate these statements, this study constructed a 5-point Likert scale, ranging from “1 = strongly disagree” to “5 = strongly agree”. This scale suggested a great level of reliability (Cronbach’s α = 0.90, M = 3.52, SD = 1.06).

#### 3.3.3. Upward Comparison

Upward comparison was evaluated using three items that were altered from previous assessments [11,57]. The items were composed of “When I read others’ news feeds or see their images, I assume they’re happy”, “When I read news feeds or see images, I assume others have a better life”, and “When I read others’ news feeds or see their images, I frequently feel envious”. All the statements were assessed on a 5-point Likert scale from 1 to 5, representing strongly disagree to strongly agree. The Cronbach’s alpha was 0.883, which indicated a good reliability (M = 3.66, SD = 1.05).

#### 3.3.4. Privacy Invasion

A total of three questions adapted from previous studies were used to assess people’s privacy invasion [58,59]. The scale comprised 3 items such as “Mobile app causes account leaks”, “Mobile app increases personal data leakage risk”, and “Personal data authorization bothers me when utilizing mobile app”. On a 5-point scale (1 = strongly disagree, 5 = strongly agree), participants rated their agreement or disagreement. These three questions were averaged to evaluate privacy invasion (Cronbach’s α = 0.895, M = 3.81, SD = 0.61).

#### 3.3.5. Emotional Exhaustion

A 7-item scale from a prior study was used to assess the extent of respondents’ emotional exhaustion [37]. The items of the scale covered, for example, “I feel emotionally exhausted by utilizing mobile app.” On a 5-point Likert scale ranging from 1 (strongly agree) to 5 (strongly disagree), respondents rated their level of agreement with each question. The scores of these four questions were then added together to provide a measurement of emotional exhaustion. With a Cronbach’s alpha of 0.941, the current scale demonstrated a great reliability (M = 3.89, SD = 0.69).

#### 3.3.6. Mobile App Fatigue

The current study updated the original response scale created by Zhang et al. for evaluating mobile app fatigue [5,32]. Some example statements consist of, “I occasionally feel exhausted while using mobile app”, and “I occasionally feel bored when using mobile app”. Participants responded to six questions by indicating their degree of agreement based on a 5-point Likert rating scale, from 1 (strongly disagree) to 5 (strongly agree). The scores were then summed to provide a measurement of mobile app fatigue (Cronbach’s α = 0.939, M = 3.87, SD = 0.62).

## 4. Data Analysis Strategy

Data was analyzed utilizing SPSS 24.0 and AMOS 25.0. SEM was employed since it is one of the most popular tools for analyzing data in the field of mobile apps. Furthermore, this approach was able to meet all of the strict prerequisites for the study.

## 5. Results

### 5.1. Demographic Characteristics

Table 1 displays the descriptive statistics of the survey data. A total of 54.9% of the participants were male and 45.1% were female, in terms of demographics. In addition, 44.0% of the respondents were between the ages of 21 and 30, while 33.8% of the respondents were between the ages of 31 and 40. In total, 46.1% had finished their undergraduate degrees, and 34.2% owned postgraduate degrees. Approximately 73.2% had more than three years of mobile app use experience. In terms of their daily duration of use, the total amount of time spent on mobile apps each day was above 3 h (65.2%). Furthermore, about 34.3% of respondents had a monthly income of 3001–6000 RMB, while 9.0% had 9001–12,000 RMB of monthly income, respectively.

### 5.2. Measurement Model Analysis

The measurement model is assessed by absolute fit indices (χ^2^/d.f. = 2.334; RMSEA = 0.051; RMR = 0.016) and the incremental fit indices (CFI = 0.946; AGFI = 0.829; IFI = 0.977; TLI = 0.939). Table 2 displays the findings, which imply an adequate model fit. According to some scholars, the main goal of measurement model analysis is to determine if each measurement item in the theory can accurately assess the latest items, including their reliability, convergent validity, and discriminant validity. The reliability was gauged with Cronbach’s alpha, composite reliability (CR), and the average variance extracted (AVE). To test the reliability of our instrument, this study analyzed the composite reliability and Cronbach’s alpha of the theoretical constructs. The Cronbach’s alpha and CR values for all the samples surpassed the desirable level of 0.7, indicating an excellent reliability. Likewise, the convergent validity was determined by the analysis of factor loadings, average variance extracted (AVE), and squared multiple correlations (SMC). The loading values ranging from 0.755 to 0.925 were above 0.7, suggesting a high convergent validity. Every construct’s AVE exceeded 0.5, indicating an acceptable convergence. The SMC values being above the recommended threshold of 0.5 indicate the suggested measurement model’s convergent validity. Table 3 shows the statistical results from the confirmatory factor analysis. In accordance with the recommendation, the calculated values of the AVE must be compared to the squared correlations of several other variables. Each AVE (diagonal terms) in Table 4 is higher than the associated squared correlation coefficients (off-diagonal terms), indicating an excellent discriminant validity. As a result, this measurement model demonstrates adequate data fitting, an acceptable reliability, and a plentiful convergent and discriminate validity.

### 5.3. Structural Model Analysis

**Figure 2:**
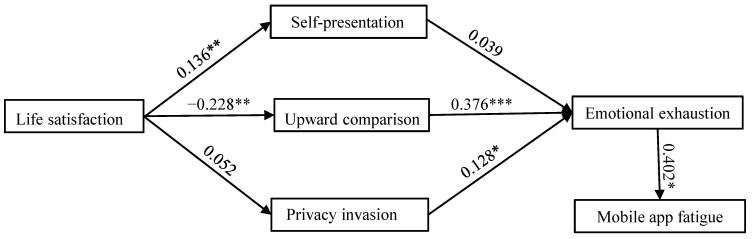
Analysis results for the structural model. Note: * *p* < 0.5; ** *p* < 0.01; *** *p* < 0.001.

The suggested structural model was integrated into AMOS software 24.0, and the structural model fit indices revealed a strong model fit (χ^2^/d.f. = 2.5903; RMSEA = 0.032; RMR = 0.016; CFI = 0.964 > 0.9; AGFI = 0.863 > 0.8; IFI = 0.989 > 0.9; and TLI = 0.958 > 0.9). Then, the investigation assessed the structural model to determine the hypothetical associations. These outcomes showed that life satisfaction positively influenced self-presentation (β = 0.136, *p* < 0.01). Meanwhile, life satisfaction negatively influenced upward comparison (β = 0.228, *p* < 0.01), thus, H1 and H2 were supported. However, life satisfaction was discovered to exert no significant or negative influence on privacy invasion (β = 0.052, *p* > 0.5). Hypothesis 3 was rejected statistically. Additionally, upward comparison (β = 0.376, *p* < 0.001) and privacy invasion (β = 0.128, *p* < 0.5) positively influenced emotional exhaustion, thus, H5 and H6 were supported. However, the results show that self-presentation was uncorrelated with emotional exhaustion, thereby, H4 was rejected. Emotional exhaustion was positively correlated with mobile app fatigue (β = 0.402, *p* < 0.5), thus, H7 was supported. The results of evaluating these hypotheses are depicted in Figure 2.

## 6. Discussion

### 6.1. Summary of Main Findings

Based on the stressor–strain–outcome theoretical framework, from the standpoint of network heterogeneity, this article concentrated on and explored the mechanism of mobile app fatigue, taking into account the impacts of life satisfaction and the different dimensions of network heterogeneity on emotional exhaustion and mobile app fatigue.

The investigation yielded some interesting findings. Firstly, it indicated that life satisfaction influences self-presentation positively. The findings are in line with the hypothesis that the psychological well-being of mobile device users has an effect on the amount of personal information they disclose [16,40,60]. As a result, a higher level of life satisfaction, or more enjoyment and contentment with mobile social platforms, is likely to have an impact on users’ network heterogeneity elements, and may boost their self-presentation participation. Meanwhile, this conclusion also supports previous studies that demonstrated a negative relationship between life satisfaction and upward comparison [1,17]. This implies that users who have a high level of happiness with their online lives on mobile app platforms are less likely to be involved in upward comparisons to support their opinions, since they could already think their social lives on these platforms are perfect. Thus, the findings suggest that individuals who believe they derive significant benefits from mobile app platforms may be protected from engaging in the potentially harmful phenomena associated with using these platforms.

However, the results show that life satisfaction is unrelated to privacy invasion. According to the results, the life satisfaction of mobile app users has no effect on privacy concerns, which is a component of network heterogeneity. This might be explained by the truth that consumers may be wary of the privacy guarantees made by online services such as Weibo and WeChat [7,18]. Such users may be proactive in maintaining privacy restrictions on these platforms in order to produce a perceivably satisfying and even perfect usage scenario, thus proactively securing their life satisfaction. However, this result may be unique to the sampled respondents, and more study is thus necessary to determine its applicability. In addition, it may be worthwhile to examine this link in the context of different types of mobile app platforms and the varying degrees of usage or content sharing that users may engage in.

Secondly, in line with previous research [1,37], the findings demonstrate that upward comparison is positively related to emotional exhaustion. This association can be attributed to the fact that mobile app users who engage in comparison constantly glance over mobile app information. As a result of this information overload, their cognitive space and information-processing abilities are burdened, resulting in psychological fatigue. As expected, the findings show that privacy invasion is positively linked with emotional exhaustion, echoing the overall tone of mobile app research. The article uncovers that there is no correlation between self-presentation and emotional exhaustion. This is a surprising conclusion that contradicts previous research. This unexpected outcome may be explained by people using social media platforms more often to connect with one another during the COVID-19 shutdown in mainland China. In these situations, users could prioritize self-disclosure, which caused them to focus more of their cognitive processing power on later-generated messages and to downplay their feelings of exhaustion. This conclusion may, however, be extremely dependent on the nature of the environmental factors (in this case, the COVID-19 pandemic) that our participants were facing.

Thirdly, the study found that upward comparison was the sole moderating factor between life satisfaction and emotional exhaustion. These findings offer support to the claim that the heterogeneity of users’ networks might assist the change of incentives or advantages, such as life satisfaction, into adverse components, such as emotional exhaustion, associated with the detrimental aspects of mobile apps [20,53]. The study argues that the mediating impact of upward comparison on the connection between life happiness and exhaustion might be related to a person’s problematic personality and features, which cause them to engage in comparisons with other users while being content with their own mobile app activities. Moreover, the study discovered that emotional exhaustion exerts positive impacts on mobile app fatigue. Consistent with previous research on the relationship between platform-generated stress and fatigue [17,29], the findings demonstrate a linkage between psychological stress and mobile app fatigue. When users’ psychological and cognitive resources are drained, they commit less time and energy to several domains, consisting of learning, life and work, and mobile devices. Therefore, mobile app fatigue is as a result of restricting the excessive use of limited psychological and cognitive resources.

### 6.2. Theoretical and Managerial Implications

This study has a number of theoretical implications. Firstly, the results add to the burgeoning literature on life satisfaction and fatigue, two topics that have hitherto been given little consideration. Furthermore, although most previous research on life satisfaction has focused on its causes, the article employs the popular SSO model to examine the effects of life satisfaction. Our results may serve as a foundation for future research that takes a multi-disciplinary approach to investigating life satisfaction and the negative effects of mobile apps, particularly fatigue among users. Secondly, although earlier research has explored the mechanics of mobile app fatigue in a variety of ways, few studies have studied this subject from the standpoint of technological stress. Users’ unfavorable psychological responses and behavioral choices are influenced by their perceived degree of technological stress. The findings of this research demonstrate that life satisfaction causes technological stress, which leads to mobile app fatigue. Third, the research extends the literature on the relationship between individuals’ mobile app usage and its negative psychosocial consequences such as exhaustion, by examining the role of network heterogeneity. The results highlight the need to examine other network heterogeneity factors, such as insufficient sleep hygiene and the spread of false news, as additional antecedents of fatigue among users.

Implications for practice are also drawn from this research. From a producer’s standpoint, it is crucial for mobile app providers to increase their user-friendly features, since life satisfaction causes the emotional exhaustion of mobile apps and disengagement from online socializing. To assist users in realizing reciprocated interaction and shared support at a level that is desirable and manageable, the designers of social media platforms ought to provide more user-friendly settings. These options might include the ability to configure notification frequency, self-disclosure filtering settings, and posting visibility settings for friends. In addition, mobile app platforms should allow users to gain mobile app literacy so that they can detect and use the customization functionalities in a proactive and fluent manner. This may effectively decrease the levels of stressors that cause emotional exhaustion, such as communication overload and privacy concerns. Furthermore, the study demonstrates that users’ life satisfaction is an antecedent of their network heterogeneity and fatigue, implying that designers, marketers, and psychologists need to comprehend the mechanisms by which mobile app users reap the advantages of this platform. Understanding the factors that contribute to life satisfaction might help stakeholders create treatments to counteract the negative effects of mobile apps that users experience.

## 7. Limitations and Implications for Future Research

Despite the fact that the research produced interesting findings, there are still several flaws that must be addressed. Firstly, the participants are primarily a young-adult segment of users in a specific geographic context, so the generalizability of this study is limited, even though the sample’s features are similar to those of general mobile app users. In the future, scholars should attempt to verify the findings by investigating larger samples from other countries, which will help generate more generalizable research results. Secondly, the current study only proves the role of upward comparison in moderating the association between life satisfaction and emotional exhaustion. It did not examine other boundary conditions, such as self-efficacy and personality, which would exert distinct influences on the model’s path of action. Further studies may include these variables in the model for a more thorough assessment. Additionally, although the study controlled demographic variables in this model, distinct factors such as gender and income, etc., may have produced different findings. Future research may take into account the various influences of demographic factors. Thirdly, the additional negative consequences of life satisfaction, such as fear of missing out, appraisal apprehension, and stalking, may occur. As a result, future research may probe the relationship between these characteristics and psychological well-being. Lastly, since the data in this present article were cross-sectional, it was difficult to determine the temporal associations between the variables in the proposed study model. Therefore, longitudinal research could be performed in subsequent studies to further prove the validity of the causal linkages between the variables demonstrated here.

## 8. Conclusions

Although mobile apps provide numerous benefits, such as enhanced information accessibility and availability of communication, they also generate considerable disadvantages, which might lead to emotional exhaustion and mobile app fatigue. In line with previous research that has attempted to emphasize the possible drawbacks of mobile app usage, this research discovered that different dimensions of network heterogeneity and life satisfaction are significant determinants of emotional exhaustion. Furthermore, upward comparison may function as a moderator in the relationship between life satisfaction and emotional exhaustion.

## Figures and Tables

**Table 1:** Summary of respondents’ demographic characteristics (N = 866).

Item	Property	Frequency	Percentage
Gender			
	Male	476	54.9
	Female	390	45.1
Age			
	Under 20 years old	170	19.6
	21–30 years old	368	44.0
	31–40 years old	293	33.8
	Above 40 years old	35	2.6
Education background			
	Middle school or lower	62	7.2
	High school	90	10.4
	Undergraduate degree	399	46.1
	Postgraduate degree	296	34.2
	Doctoral degree	19	2.1
Monthly incomes			
	Under 3000 RMB	189	21.8
	3001–6000 RMB	297	34.3
	6001–9000 RMB	276	31.9
	9001–12,000 RMB	78	9.0
	Above 12,001 RMB	26	3.0
Years of using mobile app			
	Less than 1 year	18	2.1
	1–2 years	66	7.6
	2–3 years	148	17.1
	3–4 years	245	28.3
	Above 5 years	389	44.9
Total time spent daily			
	Less than 1 h	42	4.8
	1–2 h	95	10.9
	2–3 h	165	19.1
	3–4 h	257	29.7
	Above 4 h	307	35.5

**Table 2:** Fit indices for the measurement model.

Model Fit Measures	Model Fit Criterion	Index Value	Good Model Fit (Yes/No)
Absolute fit indices			
RMSEA	<0.08	0.051	Yes
RMR	<0.05	0.016	Yes
χ^2^/d.f. (χ^2^ = 525.181, d.f. = 225)	<3	2.334	Yes
Incremental fit indices			
CFI	>0.9	0.946	Yes
AGFI	>0.8	0.829	Yes
IFI	>0.9	0.977	Yes
TLI	>0.9	0.939	Yes

**Table 3:** Statistical outcomes of confirmatory factor analysis.

Constructs and Items	Loading (>0.7)	SMC (>0.5)	CR (>0.7)	AVE (>0.5)
Life satisfaction (LA)			0.850	0.656
LA1	0.755	0.570		
LA2	0.791	0.626		
LA3	0.878	0.771		
Self-presentation (SP)			0.894	0.738
SP1	0.864	0.746		
SP2	0.839	0.704		
SP3	0.892	0.796		
Upward comparison (UC)			0.858	0.668
UC1	0.856	0.733		
UC2	0.799	0.638		
UC3	0.796	0.634		
Privacy invasion (PI)			0.899	0.749
PI1	0.788	0.621		
PI2	0.878	0.771		
PI3	0.925	0.856		
Emotional exhaustion (EN)			0.938	0.687
EN1	0.823	0.677		
EN2	0.766	0.587		
EN3	0.892	0.796		
EN4	0.779	0.607		
EN5	0.917	0.841		
EN6	0.812	0.659		
EN7	0.801	0.642		
Mobile app fatigue (MF)			0.940	0.725
MF1	0.885	0.783		
MF2	0.879	0.773		
MF3	0.769	0.568		
MF4	0.788	0.621		
MF5	0.881	0.776		
MF6	0.897	0.805		

Notes: SMC, squared multiple correlations; CR, construct reliability; and AVE, average variance extracted.

**Table 4:** Discriminant validity.

	LA	SP	UC	PI	EN	MF
LA	**0.809**					
SP	0.525 **	**0.859**				
UC	0.681 **	0.411 **	**0.817**			
PI	0.518 **	0.432 **	0.665 **	**0.865**		
EN	0.527 **	0.446 **	0.679 **	0.653 **	**0.829**	
MF	0.511 **	0.402 **	0.607 **	0.616 **	0.592 **	**0.851**

Notes: ** *p* < 0.001. Diagonal elements (bold) represent the square root of each construct’s AVE. Off-diagonal elements represent squared correlations between variables.

## Data Availability

The data of this study are available from the corresponding authors upon reasonable request.
